# Enhancement of BACE1 Activity by p25/Cdk5-Mediated Phosphorylation in Alzheimer’s Disease

**DOI:** 10.1371/journal.pone.0136950

**Published:** 2015-08-28

**Authors:** Woo-Joo Song, Mi-Young Son, Hye-Won Lee, Hyemyung Seo, Jeong Hee Kim, Sul-Hee Chung

**Affiliations:** 1 Department of Biochemistry and Molecular Biology, Neurodegeneration Control Research Center, School of Medicine, Kyung Hee University, Seoul, Korea; 2 Institute for Brain Science and Technology, Inje University, Busan, Korea; 3 Division of Molecular and Life Sciences, College of Sciences and Technology, Hanyang University, Ansan, Gyeonggi Do, Korea; 4 Department of Oral Biochemistry and Molecular Biology, School of Dentistry, Kyung Hee University, Seoul, Korea; 5 Department of Life and Nanopharmaceutical Sciences, Kyung Hee University, Seoul, Korea; University of S. Florida College of Medicine, UNITED STATES

## Abstract

The activity of beta-site amyloid precursor protein (APP) cleaving enzyme 1 (BACE1) is elevated during aging and in sporadic Alzheimer’s disease (AD), but the underlying mechanisms of this change are not well understood. p25/Cyclin-dependent kinase 5 (Cdk5) has been implicated in the pathogenesis of several neurodegenerative diseases, including AD. Here, we describe a potential mechanism by which BACE activity is increased in AD brains. First, we show that BACE1 is phosphorylated by the p25/Cdk5 complex at Thr252 and that this phosphorylation increases BACE1 activity. Then, we demonstrate that the level of phospho-BACE1 is increased in the brains of AD patients and in mammalian cells and transgenic mice that overexpress p25. Furthermore, the fraction of p25 prepared from iodixanol gradient centrifugation was unexpectedly protected by protease digestion, suggesting that p25/Cdk5-mediated BACE1 phosphorylation may occur in the lumen. These results reveal a link between p25 and BACE1 in AD brains and suggest that upregulated Cdk5 activation by p25 accelerates AD pathogenesis by enhancing BACE1 activity via phosphorylation.

## Introduction

Alzheimer’s disease (AD) is an irreversible, progressive brain disorder that is characterized by dementia. The brains of AD patients have pathological hallmarks, including amyloid plaques and neurofibrillary tangles, insoluble deposits made of proteins called Aβ and hyperphosphorylated tau, respectively. Beta-site amyloid precursor protein (APP) cleaving enzyme 1 (BACE1), a β-secretase, cleaves APP during an initial step in Aβ generation [1]. Subsequent cleavage by a γ-secretase complex including Presenilin produces Aβ. The importance of Aβ in AD pathogenesis is clear in rare familial AD cases, where mutations in the *APP* or presenilin genes produce more Aβ. However, the mechanisms underlying sporadic AD, which occurs in the majority of AD cases, are not well understood.

Accumulating evidence indicates that aberrant activation of BACE1 may play a role in the pathogenesis of sporadic AD. During aging, the most important known risk factor for AD, the enzymatic activity of BACE1 increases, although BACE1 protein levels remain unchanged [2, 3]. In the brains of sporadic AD patients, increased BACE1 activity has been consistently reported in every published study, and elevated BACE1 protein levels have also been detected in most studies [4–7]. However, some studies have also reported unchanged or decreased BACE1 levels in the AD brain [8–10], and no changes in *BACE1* mRNA expression in AD patients have been detected in most studies [5, 11–13]. Recently, increased BACE1 levels were reported only in a subgroup [~30%) of sporadic AD patients [11]. Therefore, increased BACE1 activity is not associated with similar increases in BACE1 protein or mRNA during aging and at least in a fraction of AD patients [2, 11, 14].

p25 is an aberrant cyclin-dependent kinase 5 (Cdk5) activator generated from calpain-mediated cleavage of the Cdk5 activator p35 under neurotoxic conditions, and p25/Cdk5 is a proline-directed serine/threonine kinase implicated in several neurodegenerative diseases, including AD [15]. p25 expression and Cdk5 activity are increased in the brains of sporadic AD patients [6, 16–18], although the increase in p25 expression is controversial [19]. Overexpression of p25 has been shown to increase BACE1 mRNA and protein levels via transcriptional regulation [20]. However, it is not clear how this result is associated with increased BACE1 protein and activity in AD because *BACE1* mRNA is not elevated in AD brains. Thus, although BACE1 dysregulation could play an important role in the pathogenesis of sporadic AD, the underlying mechanisms by which BACE1 activity is elevated in AD still remain unclear.

To decipher the mechanisms that underlie enhanced BACE activity observed in AD brains and during aging, we sought to determine whether the increase in p25/Cdk5 activity in AD is linked to BACE enzymatic activity via direct phosphorylation. We demonstrate that BACE1 is phosphorylated by p25/Cdk5 *in vitro* and the phosphorylated BACE1 has increased BACE1 activity. The amounts of *in vivo* phospho-BACE1 and BACE1 enzymatic activity are increased by p25 overexpression. Our results provide insights into the mechanisms of AD pathogenesis and may facilitate the development of novel drugs for the treatment of sporadic AD.

## Materials and Methods

### Proteins and antibodies

BACE1 and the p25/Cdk5 complex were from Invitrogen (Carlsbad, USA) and Upstate Biotechnology (Lake Placid, USA) respectively. p25 and Cdk5 antibodies were obtained from Santa Cruz Biotechnology (Santa Cruz, USA); APP and phospho-APP antibodies, from Cell Signaling Technology (Danvers, USA); anti-actin antibodies, from Sigma (St. Louis, USA); anti-BACE1 antibodies to the C-terminal peptide, from Chemicon International (Temecula USA), Calbiochem (Billerica, USA), and Santa Cruz;, and the peptide 296–310 (RLPKKVFEAAVKSIK) was custom-made (Peptron, Korea). A phosphospecific BACE1 (P-BACE1) antibody was raised against a synthetic phosphopeptide [SLWYThr^252^(PO_4_)PIRR] and affinity-purified first with a cognate nonphosphopeptide affinity column and then with a phosphopeptide column (Peptron).

### Cdk5 kinase assay

For autoradiography analysis, purified BACE1 protein (1.5 μg) was incubated for 1 h at 30°C with the p25/Cdk5 complex (40 ng) in kinase buffer (20 mM MOPS, pH 7.0, 10 mM MgCl_2_, 1 mM DTT, 20 μM sodium orthovanadate) containing 100 μM cold ATP and 5 μCi [γ-^32^P]ATP. The reaction mixtures were separated on SDS-polyacrylamide gels. For analysis by immunoblotting with a P-BACE1 antibody, BACE1 protein (0.32 μg) was incubated with the recombinant p25/Cdk5 complex (50 ng) in kinase buffer containing 200 μM ATP at 30°C for 2 h.

### BACE1 activity and Aβ assays

For the BACE1 enzymatic activity assay, cell culture or brain lysates were incubated with 10 μM fluorogenic peptide substrate IV, which contains an APPsw β-secretase cleavage site, in 96-well plates, and ELISA was performed according to the manufacturer’s recommendations (R&D Systems, USA). Fluorescence was measured at an excitation wavelength of 320 nm and an emission wavelength of 405 nm using a SPECTRAmax Gemini XS fluorometer with Softmax PRO 4.3 LS software (Molecular Devices, USA). The amount of Aβ peptide was measured using a sandwich enzyme-linked immunosorbent assay (ELISA) kit according to the manufacturer’s recommendations (IBL, Japan). For the measurement of Aβ in the brain, SDS (2%)-extracted hippocampal lysates were used [21]. The amount of Aβ was normalized to the amount of total protein.

### DNA constructs, transfections, cell culture and secretion assay

Full-length p25, Cdk5, and BACE1 were cloned from a human brain cDNA library using reverse transcription polymerase chain reaction (RT-PCR). The amplified DNA fragments were then subcloned into either the pcDNA3.1myc/his or the pTRE-pur vector (Invitrogen). The human BACE1 T252A construct was generated by DpnI-mediated site-directed mutagenesis, and the sequences of the resulting clones were verified. HEK293T and SK-N-BE(2)C cells were cultured at 37°C and 5% CO_2_ in Dulbecco’s modified Eagle medium (DMEM) or DMEM/F-12 medium (Gibco-BRL, USA), respectively, supplemented with 10% fetal bovine serum (FBS). PC12(Tet-Off) cells were cultured at 37°C and 5% CO_2_ in RPMI1640 medium supplemented with 10% FBS, 5% horse serum (HS), and 100 μg/mL G418. Cells were transiently or stably transfected using Lipofectamine 2000 (Invitrogen) following the manufacturer’s protocol. PC12(Tet-Off) cells stably expressing p25 were generated as described previously [22]. The human growth hormone (hGH) secretion assay was performed in PC12 cells (ATCC), as previously described [22, 23].

### Preparation of lysates from cell cultures and brains

HEK293T and SK-N-BE(2)C cells (ATCC) were harvested 24 h after transient transfection. To induce p25 expression, PC12 cells stably transfected with p25 [22] were incubated for 3 days without doxycycline. For roscovitine (50 μM) treatment or for the Aβ assay, PC12 cells stably transfected with p25 were incubated for an additional 24 h on new culture plates with fresh conditioned medium (RPMI640, 5% HS, 1% FBS; with or without roscovitine). The amount of Aβ peptide secreted into the conditioned media was measured as described above. Cells were then lysed in RIPA buffer (50 mM Tris, pH 8.0, 150 mM NaCl, 1% NP-40, 0.1% SDS, 0.5% deoxycholic acid) containing 1 mM PMSF and a protease inhibitor cocktail.

p25 transgenic (TG) mice, which overexpress the human *p25* gene under the control of the neuron-specific enolase (NSE) promoter [24], were obtained from Jackson Labs and subsequently inbred. TG mice and control littermates were sacrificed by cervical dislocation. Brains were dissected, and snap-frozen in liquid nitrogen. Dounce homogenization was performed in RIPA buffer containing 1 mM PMSF, a protease inhibitor cocktail, and phosphatase inhibitors (50 mM NaF, 1 mM Na orthovanadate). Experiments were performed in accordance with guidelines of the Inje University Council Directive for the proper care and use of laboratory animals. This animal care and use protocol was reviewed and approved by the Institutional Animal Care and Use Committee (IACUC) at College of Medicine Inje University.

Postmortem brain samples from AD patients and normal controls were provided by the Harvard Brain Bank at McLean Hospital, and the brain lysates were prepared as described previously [25]. Lysates were either used fresh or were aliquoted, frozen in liquid nitrogen, and stored at -80°C. Typically, 50 μg of total protein was used for western blotting, BACE1 activity assay, and Aβ assay. Unless indicated otherwise, BACE1 bands corresponding to the molecular size of phospho-BACE1 are shown in western blots. Co-immunoprecipitation using cdk5 antibody was performed as described previously [26].

### Immunohistochemistry

Mice were anesthetized with isoflurane and intracardially perfused with phosphate-buffered saline (PBS), followed by 4% paraformaldehyde. Brains were fixed in 4% paraformaldehyde in PBS overnight at 4°C and were then cryoprotected. Sections (16-μm thick) of mouse brains were prepared as previously described [27] and used for immunofluorescence microscopy after incubation with P-BACE1 antibodies (1:500 dilution) and Cy3-conjugated goat anti-rabbit antibodies.

### Iodixanol step gradient

Mouse brain organelles were separated by iodixanol step gradient, as described by Lee *et al*. [28]. Hippocampus tissue from 19-month-old p25 TG mice was homogenized in 1 mL homogenization buffer (250 mM sucrose, 20 mM Tris-HCl, pH 7.4, 1 mM EGTA, 1 mM EDTA, and protease and phosphatase inhibitors). Brain homogenates were centrifuged to obtain the postnuclear supernatant (PNS). The PNS was then diluted to 25% OptiPrep (Axis-Shield PoC.) using 50% OptiPrep in homogenization buffer and placed at the bottom of an ultracentrifuge tube. This mixture was then overlaid successively with 1 mL each of 20%, 18.5%, 16.5%, 14.5%, 12.5%, 10.5%, 8.5%, 6.5%, and 5% OptiPrep in cold homogenization buffer. The gradient was centrifuged for 20 h at 27,000 rpm at 4°C. Fractions of 1 mL each were collected from the top of the gradient and analyzed by western blotting with antibodies as indicated.

### Protease-protection assay

Protease-protection assays were performed to determine protein topology using a modified method described by Lin *et al*. [29]. For proteinase K (PK) digestion, aliquots of iodixanol gradient fractions 5 and 10 of hippocampus samples from 19-month-old p25 TG mice were suspended in 25 μL reaction buffer containing 10 mM Tris-HCl (pH 7.8), 150 mM KCl, 2 mM MgCl_2_, and 2 mM CaCl_2_, followed by incubation with or without 50 or 100 μg PK (Boehringer Mannheim GmbH, Germany) in the presence or absence of Triton X-100 (TX-100) at room temperature for 30 min. Thereafter, 10 mM PMSF was added to stop the reaction. Proteins were analyzed by western blotting with appropriate antibodies.

### Statistical analysis

All numerical data are presented as mean ± SEM. Comparison between two groups was carried out using two-tailed Student’s t-tests except the analysis of control and AD groups which was performed by Mann-Whitney U test. A p-value of < 0.05 was considered statistically significant.

## Results

### BACE1 is phosphorylated by p25/Cdk5 at Thr252

**Fig 1:**
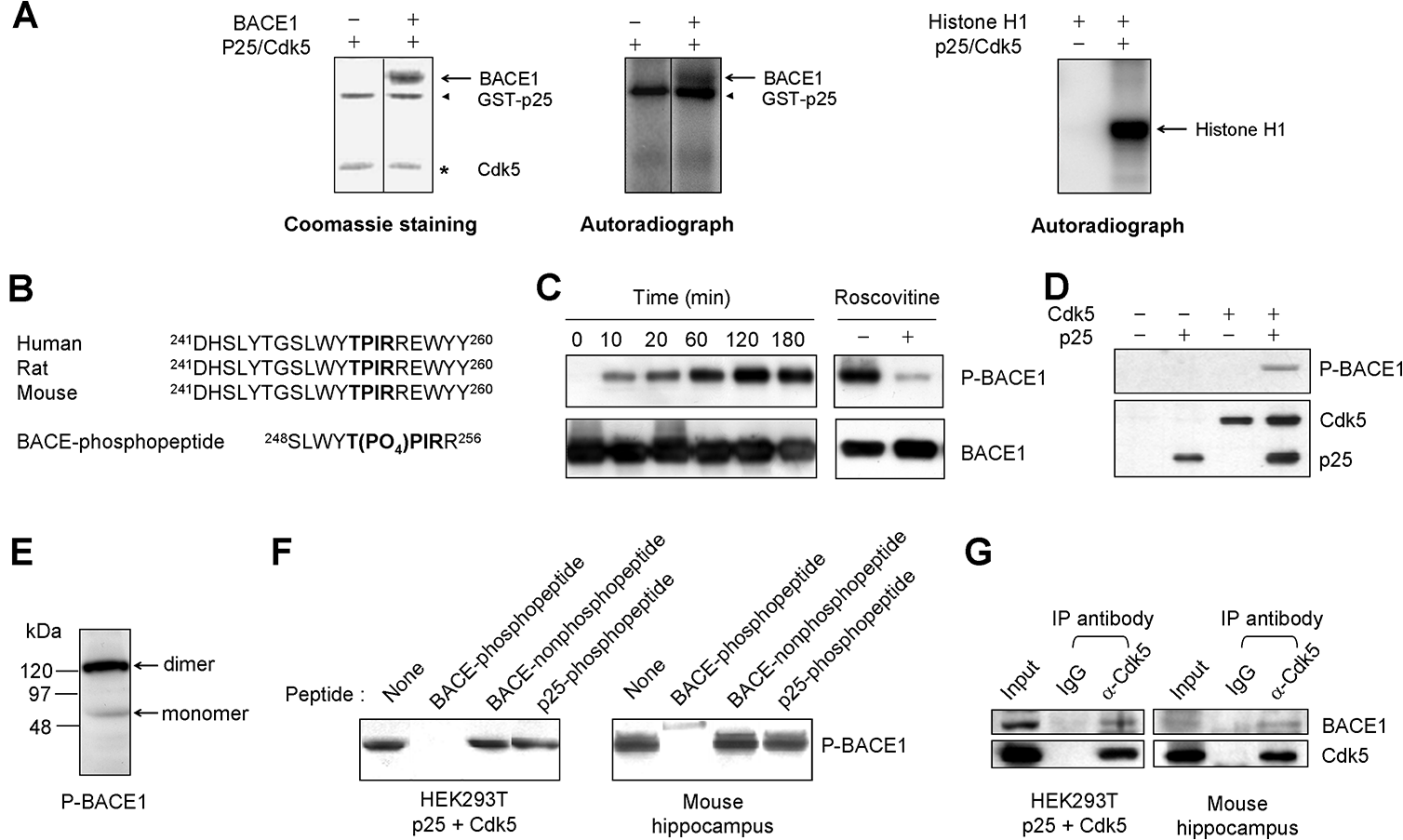
Phosphorylation of BACE1 at Thr252 by p25/Cdk5. A. Coomassie staining (left) and autoradiograph (middle) of SDS polyacrylamide gels containing the products of *in vitro* kinase assays that employed a BACE1 substrate and the p25/Cdk5 complex. Phospho-BACE1 and phospho-p25 are indicated by an arrow and an arrowhead, respectively. Histone H1, a known substrate of p25/Cdk5, was used as a positive control (right). B. Alignment of human, rat, and mouse BACE1 amino acid sequences. The consensus Cdk5 phosphorylation site is marked in bold. The phosphopeptide used to generate a phosphospecific BACE1 antibody is shown. C. Products of *in vitro* kinase assays that employed a BACE1 substrate and the p25/Cdk5 complex. Reactions were incubated for the indicated times (left panel) and treated with or without roscovitine, a Cdk5 inhibitor (right panel). Products were then analyzed by western blotting with phosphospecific BACE1 (P-BACE1) antibodies and anti-BACE1 antibodies. D. Western blots for detection of P-BACE1 and BACE1 from HEK293T cells transfected with plasmids encoding the indicated proteins. E. Rat brain lysates were analyzed by western blotting with anti-P-BACE1 antibodies. F. **Peptide competition assays demonstrated the specificity of anti-P-BACE1 antibodies.** Lysates from HEK293T cells transiently transfected with plasmids encoding p25 and Cdk5 (left panel) or mouse hippocampal lysates (right panel) were analyzed by western blotting with anti-P-BACE1 antibodies pre-incubated in the absence (None) or presence of BACE1-phosphopeptide (SLWYT(PO_4_)PIRR), BACE1-nonphosphopeptide (SLWYTPIRR), or p25-phosphopeptide (SAGT(PO_4_)PKRVI). G. Co-immunoprecipitation assays showed the interaction between BACE1 and Cdk5. HEK293T cell lysates that were transfected with plasmids encoding p25 and Cdk5 (left panel) or p25 TG mouse hippocampal lysates (right panel) were immunoprecipitated with control IgG or anti-Cdk5 antibodies and then subjected to immunoblot analysis with the indicated antibodies.

To determine whether the p25/Cdk5 complex can phosphorylate BACE1 *in vitro*, we incubated purified BACE1 with recombinant p25/Cdk5 in a kinase assay buffer containing [γ-^32^P]ATP. A band corresponding to the molecular size of BACE1 was detected by autoradiography only when both BACE1 and p25/Cdk5 were present in the reaction mixture (Fig 1A). This indicates that BACE1 is phosphorylated by p25/Cdk5. However, the signal of the phospho-BACE1 band was weak, suggesting that a large fraction of the insect cell-derived BACE1 may not have folded properly and thus may have been unable to serve as a substrate for p25/Cdk5. In agreement with a previous report [30], we also detected phosphorylation of p25 by Cdk5 (Fig 1A).

**Fig 2:**
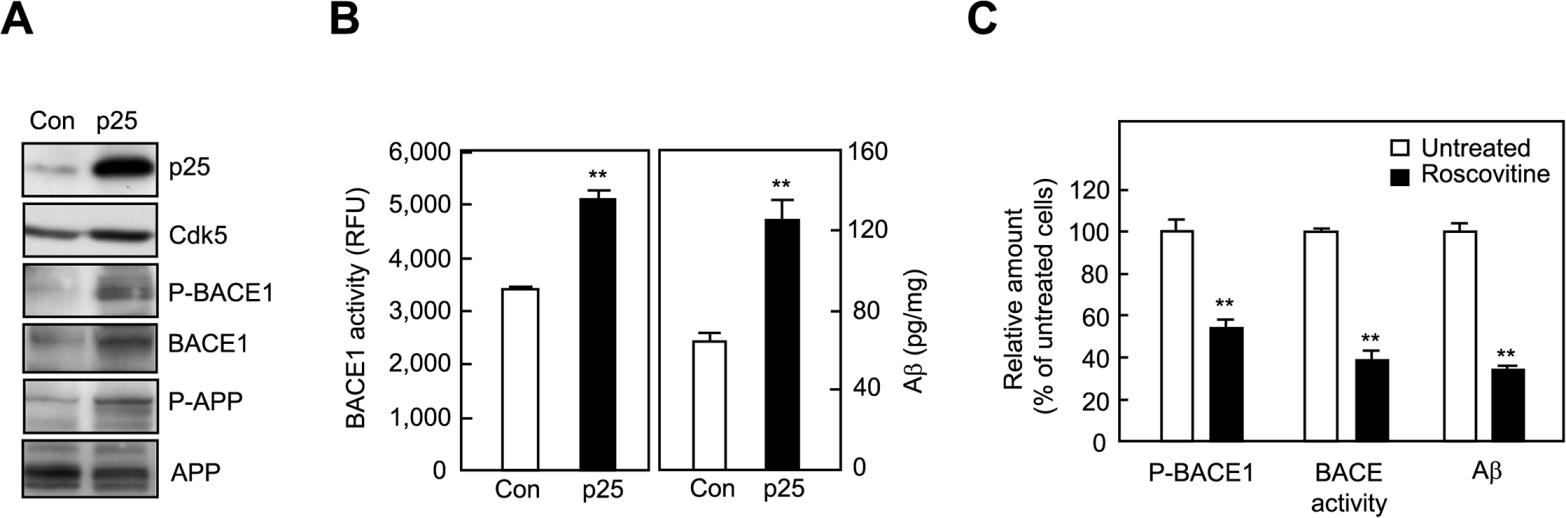
Phospho-BACE1 and BACE activity are increased in p25-overexpressing cells. A. Representative western blots of PC12(Tet-Off) cells (Con) and PC12(Tet-Off) cells stably transfected with p25 (p25). B. BACE activity and the amount of Aβ secreted into the medium by PC12(Tet-Off) cells and cells stably transfected with p25. C. Effects of roscovitine on P-BACE1, BACE activity, and Aβ secretion in cells stably transfected with p25. Data in B and C are means ± SEMs and were obtained from 2–4 independent experiments performed in triplicate. **p < 0.01 versus the control by 2-tailed Student’s t-test.

**Fig 3:**
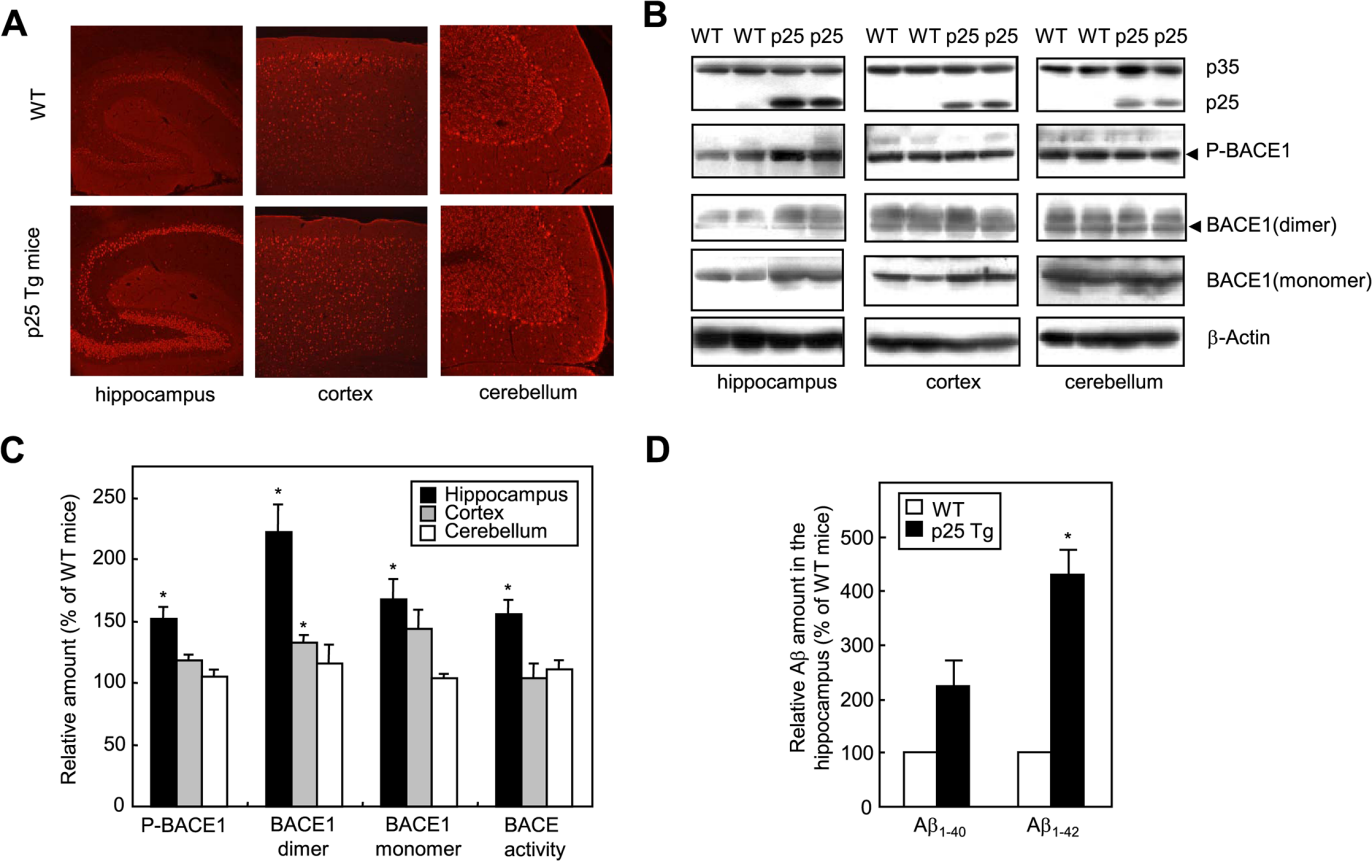
Phospho-BACE1 is markedly increased in the hippocampus of p25 transgenic (TG) mice. A. Immunohistochemistry of brain sections from p25 TG mice (20 weeks old, lower panel) and age-matched wild-type (WT) mice (upper panel) using anti-P-BACE1 antibodies. B. Representative western blots of indicated brain regions in two p25 TG mice and control littermates. Arrowheads indicate phospho-BACE1 and the corresponding BACE1 dimer. C. Densitometric analysis of western blots (normalized by β-actin signal) and BACE activity in p25 TG mice plotted as a percent of the WT. D. Aβ amounts in the hippocampus of p25 TG mice plotted as a percent of the WT. Data in C and D are means ± SEMs of 4–7 independent experiments. *p < 0.05 versus WT mice by 2-tailed Student’s t-test.

**Fig 4:**
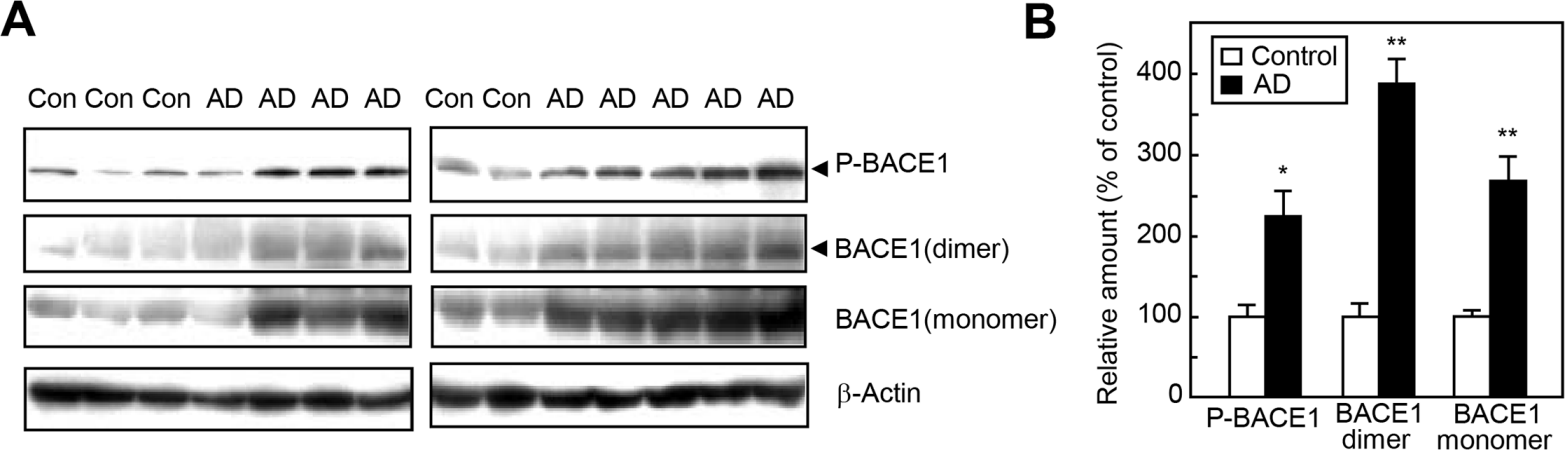
Phospho-BACE1 protein is elevated in the superior frontal cortex of human AD brains. A. Representative western blots of superior frontal cortex tissue (Brodmann area 9) from human AD patients (AD) and normal controls (Con). Arrowheads indicate phospho-BACE1 and the corresponding BACE1 dimer. B. Densitometric analysis of phospho-BACE1 and BACE1 signals in the western blot shown in A, normalized by β-actin signals. Amounts of phospho-BACE1 and BACE1 are plotted as a percent of the control. Control and AD groups consisted of 5 normal (mean age 65 ± 3 years; postmortem interval [PMI], 19 ± 1 h) and 10 AD patients (mean age 69 ± 2 years; PMI, 18 ± 2 h), respectively. *p < 0.05, **p < 0.01 versus the control by Mann-Whitney U test.

An examination of the amino acid sequence of BACE1 revealed the presence of a consensus Cdk5 phosphorylation site at Thr252, which was followed by proline and basic amino acids that are conserved in humans, rats, and mice (Fig 1B). To determine whether Cdk5 phosphorylated BACE1 at Thr252, we used a custom-made rabbit polyclonal antibody to the phosphopeptide SLWYT^252^(PO_4_)PIRR (P-BACE1 antibody) (Fig 1B). To determine the specificity of the P-BACE1 antibody, purified recombinant BACE1 was incubated with p25/Cdk5 *in vitro* for various times. The P-BACE1 antibody detected a time-dependent increase in BACE1 phosphorylation, which was strongly reduced in the presence of a Cdk5 inhibitor, roscovitine (Fig 1C), indicating that the antibody detected the phosphorylated form of BACE1. Next, we performed western blotting with lysates from HEK293T cells transiently transfected with expression plasmids encoding p25, Cdk5, or both. The P-BACE1 antibody detected a band corresponding to the size of endogenous BACE1 only in the presence of both p25 and Cdk5 (Fig 1D). In mouse and rat brains, a 120–130 kDa band corresponding to the size of the BACE1 dimer was predominantly detected by the P-BACE1 antibody (Fig 1E). This was unexpected because most studies have suggested that the BACE1 dimer is not the major form. The BACE1 dimer band was also detected by an anti-BACE1 polyclonal antibody targeting the C-terminal region of BACE1 (Figs 2, 3 and 4). In fact, the 60-kDa band corresponding to the BACE1 monomer size was difficult to detect with the P-BACE1 antibody in most western blots of mouse brain lysates. An SDS-resistant dimer band (∼110 kDa) was also observed by immunoblotting when purified recombinant BACE1 (∼55 kDa) was phosphorylated *in vitro* by p25/Cdk5 (unpublished observation). The BACE1 dimer was resistant to nonionic detergents and reducing and denaturing conditions, such as boiling in SDS, which suggests the existence of strong intermolecular interactions of BACE1 proteins. These results were consistent with previous reports on SDS- and boiling-resistant BACE1 dimers [31–33] and suggest that BACE1 may form a covalently modified, non–disulfide-linked homodimer through post-translational modification, such as phosphorylation of BACE1 at Thr252 by p25/Cdk5. The specificity of the P-BACE1 antibody was further confirmed by peptide competition experiments showing that the antibody signal in the western blot was blocked by pre-incubation with the BACE1 phosphopeptide (Fig 1F).

To explore the interaction between BACE1 and p25/Cdk5 proteins, co-immunoprecipitation assays were performed with the lysates of HEK293T cells transfected with plasmids encoding p25 and Cdk5. BACE1 was coimmunoprecipitated with Cdk5 (Fig 1G, left panel). Furthermore, BACE1 was immunoprecipitated with the anti-Cdk5 antibody from p25 TG mouse hippocampal lysates (Fig 1G, right panel), suggesting the interaction between endogenous BACE1 and Cdk5.

### Phospho-BACE1 and BACE activity are increased in p25-overexpressing cells and TG mice

To analyze the interplay between p25 and BACE1, and to avoid potential toxicity from p25 overexpression [16], we generated stably transfected PC12 cells in which exogenous p25 expression was regulated by a Tet-Off system. Consistent with a previous report demonstrating that p25 overexpression is associated with increased BACE1 mRNA, protein, and activity in human neuroblastoma SH-SY5Y cells [20], BACE1 protein levels, BACE1 activity, and Aβ secreted in the medium were increased substantially in PC12 cells stably transfected with p25 (Fig 2A and 2B). The amount of phospho-BACE1 was increased in p25-transfected cells compared to control cells (Fig 2A). Phospho-Thr668-APP, a form of APP subjected to p25/Cdk5-mediated phosphorylation [28, 34], was also increased in p25-transfected cells. Furthermore, BACE1 phosphorylation, BACE1 activity, and Aβ secretion were reduced by roscovitine (Fig 2C), suggesting a link between p25 expression and BACE activity through Cdk5-mediated phosphorylation.

To confirm the above findings, we analyzed brains of p25 TG mice. Immunohistochemical analyses revealed that BACE1 phosphorylation was markedly increased in the hippocampus of p25 TG mice compared to that of control littermates (Fig 3A). The results were corroborated by western blotting with brain lysates prepared from the hippocampus, cortex, and cerebellum of p25 TG mice and control littermates (Fig 3B). The p25 TG mice showed robust p25 expression in the hippocampus and less p25 expression in the cerebellum (Fig 3B). Phospho-BACE1 was increased by 52% in the hippocampus of p25 TG mice as compared to control littermates (Fig 3C). The P-BACE1 antibody detected a 120–130 kDa protein band corresponding to the BACE1 dimer size [31, 35]. Western blotting revealed that the amounts of the BACE1 dimer and the BACE1 monomer (60–65 kDa) were also increased by 122% and 68%, respectively, in the hippocampus of p25 TG mice (Fig 3B and 3C). In agreement with a previous report [20], BACE1 enzymatic activity was increased by 56% in the hippocampus of p25 TG mice (Fig 3C). Furthermore, the amounts of Aβ_1–40_ and Aβ_1–42_ in the hippocampus of p25 TG mice were increased by 2.2- and 4.3-fold, respectively, compared to control littermates (Fig 3D). Taken together, these results demonstrate that BACE1 phosphorylation, BACE activity, and Aβ levels are enhanced with p25 overexpression.

### BACE1 phosphorylation is elevated in human AD brains

Both senile plaques and neurofibrillary tangles are found in the frontal cortex and hippocampus of AD brains [4]. To investigate the degree of phosphorylation of BACE1 in sporadic AD patients, we performed western blot analyses on superior frontal cortex samples from AD patients and age-matched controls. Consistent with previous findings [5, 7], we observed increased levels of the BACE1 monomer (Fig 4A and 4B) in AD brains compared to controls. We found that the amounts of phospho-BACE1 and the corresponding BACE1 dimer in AD brain tissue were significantly increased compared to controls (Fig 4A and 4B). These results suggest that elevated BACE1 phosphorylation is linked to the pathogenesis of sporadic AD.

### p25/Cdk5-mediated phosphorylation of BACE1 increases BACE1 activity

**Fig 5:**
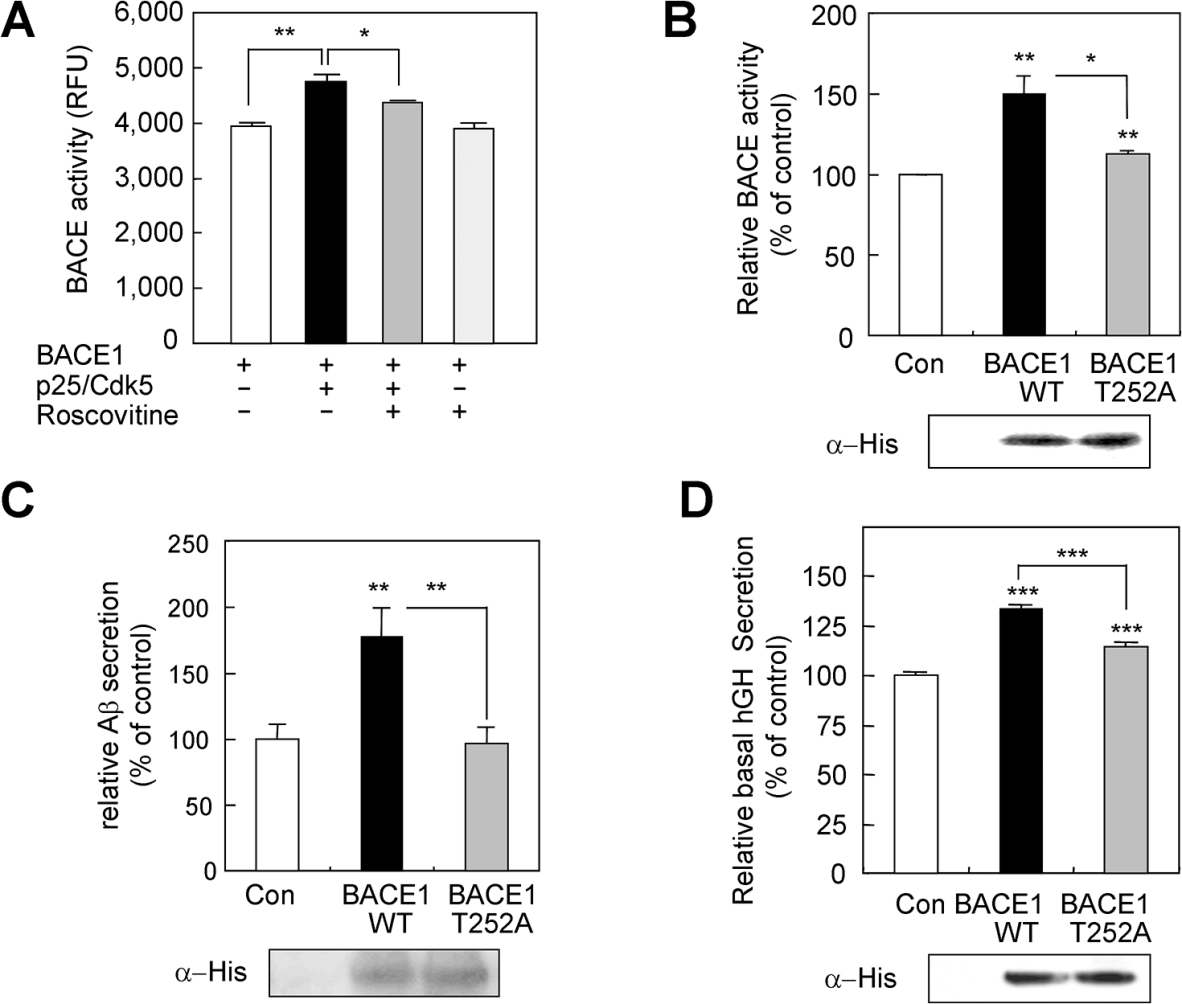
p25/Cdk5-mediated phosphorylation of BACE1 increases its activity. A. Effect of *in vitro* p25/Cdk5-mediated phosphorylation of BACE1 on its enzymatic activity. B. Effect of a phosphorylation-defective BACE1 mutant (BACE1 T252A) on BACE activity in SK-N-BE(2)C cells. C. Effect of BACE1 T252A on the production of Aβ in HEK293 cells stably expressing APPsw. D. Effect of phosphorylation of Thr252 in BACE1 on basal hGH secretion in PC12 cells. Basal secretion from BACE1 or BACE1 T252A-transfected cells is plotted as a percentage of the secretion from PC12 cells transfected with the control plasmid. Representative immunoblot of extracts from transfected cells with an anti-His antibody is shown below the corresponding graph.

The facts that the BACE1 dimer is more active than the monomer [33, 35] and that the Thr252 phosphorylation site is close to the active site of BACE1 [36] prompted us to examine the effect of phosphorylation on BACE1 enzymatic activity *in vitro*. The activity of BACE1 that had been phosphorylated by p25/Cdk5 *in vitro* was increased by 20% when compared with that of unphosphorylated BACE1 (Fig 5A). This rather small increase is likely related to the low level of *in vitro* BACE1 phosphorylation by p25/Cdk5 (Fig 1A). Roscovitine, a Cdk5 inhibitor, reduced BACE1 phosphorylation substantially (Figs 1B and 2C) and inhibited BACE1 activity compared to that of phosphorylated BACE1 (Fig 5A). However, roscovitine did not have any effect on BACE1 activity in the absence of p25/Cdk5 (Fig 5A). These results demonstrate that *in vitro* phosphorylation of BACE1 by p25/Cdk5 increases BACE1 activity.

To further assess the role of Thr252 phosphorylation in BACE1 enzymatic activity, SK-N-BE(2)C human neuroblastoma cells were transfected with either a control plasmid or with plasmids encoding full-length wild-type BACE1 (WT) and a phosphorylation-defective BACE1 mutant (T252A). The expression of WT BACE1 and BACE1T252A enhanced the BACE1 activity by 50% ± 10% (*p* < 0.01 versus control, n = 6) and 13% ± 2% (*p* < 0.01 versus control, n = 6), respectively (Fig 5B). The BACE1 activity in cell cultures expressing BACE1T252A was significantly reduced compared with that expressing WT BACE1 (Fig 5B). We then examined the effects of Thr252 phosphorylation on the production of Aβ using stable HEK293 cell lines overexpressing APPsw. Transfection of HEK293 cells stably expressing APPsw with WT BACE1 increased Aβ levels by 77% ± 21% (*p* < 0.01 versus control, n = 6), whereas transient expression of the T252A mutant had little effect on the amount of Aβ (Fig 5C). Similar amounts of BACE1 WT and the BACE1(T252A) mutant were used for the BACE1 activity and Aβ assays (Fig 5B and 5C). These results demonstrate that Thr252 phosphorylation site is important for BACE1 activity.

We previously reported that BACE1 overexpression stimulates basal secretion in PC12 cells [37], suggesting that elevated BACE1 in AD brains may contribute to the altered neurotransmitter pathology of AD. We also showed that expression of p25 causes an increase in basal secretion, and co-expression of BACE1 and p25 stimulates basal secretion in an additive manner in PC12 cells [22]. To determine the role of Thr252 phosphorylation in the BACE1-induced increase in basal secretion, the effects of WT BACE1 or the T252A mutant on basal hGH secretion were compared in PC12 cells. The expression of WT BACE1 and BACE1T252A increased basal hGH secretion by 33% ± 3% (*p* < 0.001 versus control, n = 9) and 15% ± 2% (*p* < 0.001 versus control, n = 9) respectively (Fig 5D). The basal secretion of hGH in cell cultures expressing BACE1T252A was significantly decreased compared with that expressing WT BACE1 (Fig 5D), while the expression levels of BACE1 WT and BACE1(T252A) mutant were similar, suggesting that Thr252 phosphorylation played a role in the BACE1-induced elevation in basal secretion in PC12 cells. Taken together, these results show the importance of Thr252 phosphorylation for the activity and function of BACE1.

### Phospho-BACE1 is cofractionated with p25 and endosome markers in an iodixanol gradient, and fraction 5 of p25 is present in the lumen

**Fig 6:**
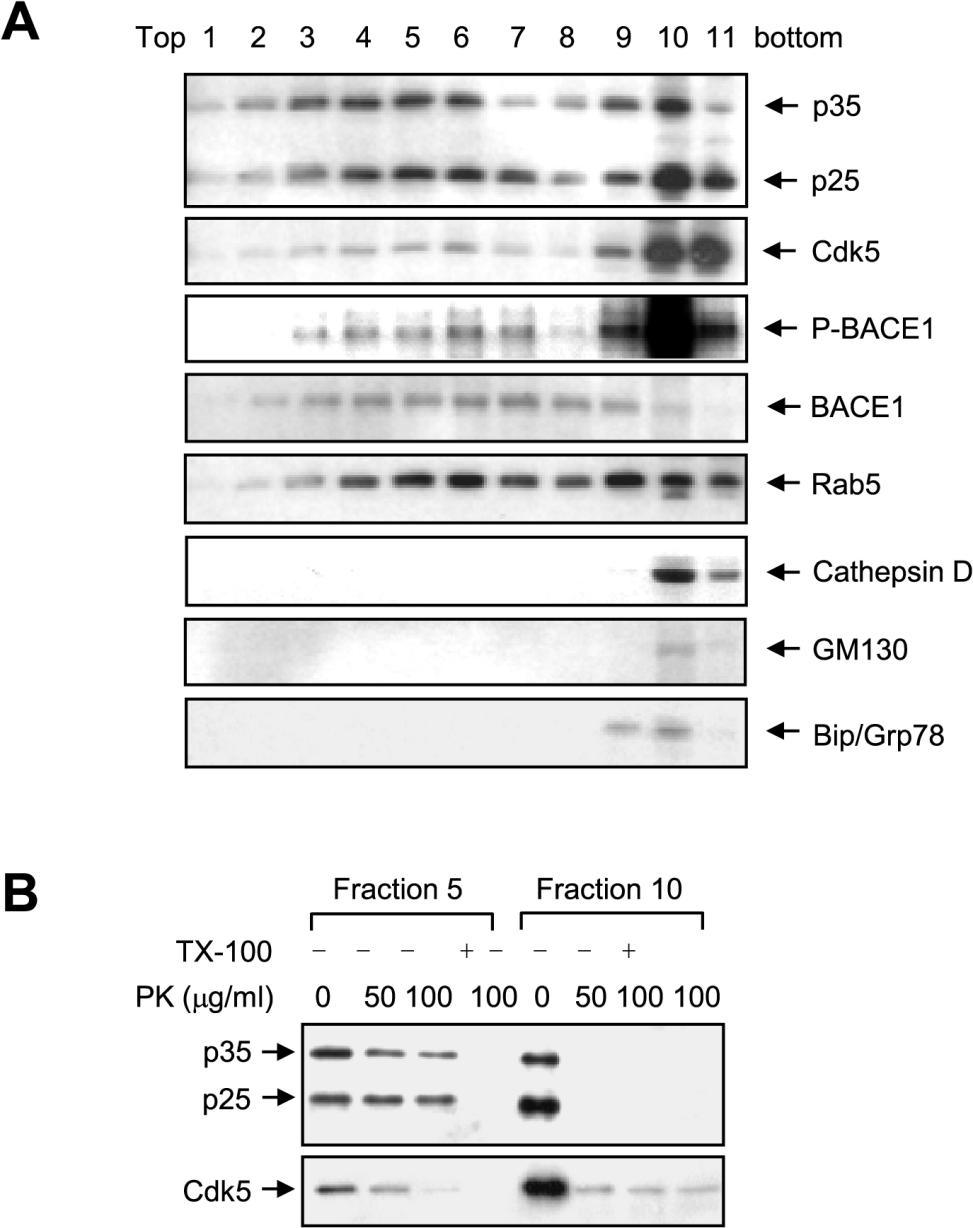
Phospho-BACE1 is cofractionated with p25 and endosome marker in an iodixanol gradient, and fraction 5 of p25 is a luminal protein. A. Nineteen-month-old p25 TG mouse hippocampus homogenates were separated through an iodixanol step gradient. Western blot analysis showed the distribution of the indicated proteins and organelle markers: Rab5 (early endosome), cathepsin D (lysosome), GM130 (Golgi apparatus), and Bip/Grp78 (ER). B. Two selected fractions, 5 and 10, were digested with PK in the presence or absence of the detergent Triton X-100 (TX-100) followed by immunoblotting analysis with indicated antibodies.

BACE1 is mainly localized within acidic compartments, such as the endosome, a major compartment for APP processing, and its active site is in the lumen of the vesicles [14, 28, 36, 38]. p25 is a cytosolic protein; however, the Thr252 phosphorylation site of BACE1 faces either the luminal or extracellular side. To understand the mechanism by which p25/Cdk5 phosphorylates BACE1 in a different compartment, various organelles from hippocampal tissues of 19-month-old p25 TG mice were separated on an iodixanol (Optiprep) density gradient. The distribution of organelles in the different fractions was monitored for colocalization with known organelle markers. Western blotting using the P-BACE1 antibody demonstrated a broad distribution of P-BACE1 between fractions 3–7 and 9–11 (Fig 6A). BACE1 had a wide distribution between fractions 2–10 (Fig 6A). About ~90% of loaded proteins were found in the bottom fractions 10–11. Lysosomes (cathepsin D), Golgi apparatus (GM130) and endoplasmic reticulum (ER; Bip/Grp78) were segregated to the bottom of the gradient (Fig 6A). Some of the P-BACE1 in the bottom gradients may represent unseparated or aggregated lysates or immature P-BACE1 in the secretory pathway. The fractionation profiles of p25 and P-BACE1 mostly overlapped with that of Rab5, an early endosome marker (Fig 6A), suggesting that the p25/Cdk5-mediated phosphorylation of BACE1 may occur in Rab5-positive endosomes.

To examine the topological properties of p25 and related proteins, 2 representative fractions, 5 and 10, were selected and digested with proteinase K (PK) in the presence or absence of detergent, followed by immunoblotting analysis (Fig 6B). Surprisingly, p25 in fraction 5 was resistant to PK digestion at 100 μg/mL, and a portion of p35 and Cdk5 were also resistant to PK digestion (Fig 6B), while the proteins in fraction 5 were digested in the presence of detergent (1% Triton X-100). However, the proteins of interest in fraction 10 were almost completely digested by PK (Fig 6B). These results indicate that p25 and a portion of p35 and Cdk5 in fraction 5 are protected from protease digestion because they are present in the lumen of vesicles and that p25/Cdk5-mediated phosphorylation of BACE1 may occur in the lumen of endosomes.

## Discussion

In this study, we demonstrate for the first time that BACE1 is phosphorylated by Cdk5. We conclude that BACE1 phosphorylation by p25/Cdk5 contributes to the observed increase in BACE activity based on the following data: (1) *in vitro* experiments using purified BACE1 and p25/Cdk5 provide direct evidence that phospho-BACE1 increases BACE1 activity; (2) the BACE1T252A mutant inhibits BACE1 activity and basal secretion in cells compared to that of WT BACE1, suggesting that Thr252 phosphorylation is important for BACE1 activity/function; and (3) BACE1 phosphorylation, BACE activity, and Aβ levels are enhanced in p25-overexpressing cells, p25 TG mice, and human AD brains, supporting a link between phospho-BACE1 and BACE1 activity. BACE1 activity, but not the amount of BACE1, increases in humans, monkeys, and mice during aging, the major risk factor for AD [2, 3]. In AD brains, BACE1 activity is disproportionately increased relative to BACE1 protein or mRNA levels [4, 14]. Perhaps multiple factors, including transcriptional, post-transcriptional, and/or post-translational regulation of BACE1, may contribute to the elevated expression and activity of BACE1 [39]. The phosphorylation of BACE1 by p25/Cdk5 described in this study could be one mechanism by which BACE1 activity is increased without changes in BACE1 protein levels.

The inconsistencies in reports of p25 levels in AD brain samples [16, 17, 19] are likely due to the short half-life of p25 [15] as well as variations in human brain samples and treatment conditions (e.g., postmortem interval, number of freezing/thaw cycles, etc.). Recent studies show that Cdk5 protein levels and the ratio of p25:p35 are increased in AD brains [6]. Cdk5 is also increased in the brains of mild cognitive impairment (MCI) patients, who show a higher risk of AD [40]. It is important to note that we observed increases in phospho-BACE1 and BACE1 activity as well as increased amounts of p25 in normal aging (unpublished observation), suggesting that p25/Cdk5-mediated BACE1 phosphorylation may also explain the age-related increase in BACE1 activity. In fact, the discrepancy in p25 expression in AD does not necessarily contradict the importance of the p25/Cdk5 mechanism for phosphorylating BACE1 and increasing BACE1 activity, as described in this study. The recent finding that partial genetic deletion of BACE1 in p25 TG mice reduced Aβ levels and rescued synaptic and cognitive deficits seen in p25 TG mice [41] provides *in vivo* proof for the link between BACE1 activity and p25 protein. Further investigations measuring BACE1 activity in BACE1 Thr252A knock-in p25 TG mice may further substantiate the functional importance of this phosphorylation event.

Previous studies have shown that miscompartmentalization occurs in patients with AD, including mislocalization of extracellular heparan sulfate to the cytoplasmic compartment [42, 43]; however, the underlying mechanisms are poorly understood. Neuronal endocytic abnormalities, including endosomal enlargement, are early pathological alterations in AD that contribute to increased Aβ generation [44, 45]. BACE1 levels are enriched in enlarged endosomes, and BACE1 is colocalized with phospho-Thr668-APP in these compartments [28]. The predicted structure of BACE1 suggests that the location of the BACE1 phosphorylation site at Thr252 is in the lumen of the organelle or on the external surface of the plasma membrane. This is not easily reconciled with the idea that p25/Cdk5, which is localized in the cytosol, participates in the phosphorylation of BACE1. Biochemical fractionation of organelles and protease protection assays suggested that a fraction of p25 is present in the lumen. Although the location and the detailed mechanism by which p25/Cdk5 phosphorylates BACE1 need further investigation, our results may provide the foundation for future studies on this topic. We propose several models of p25/Cdk5-mediated BACE1 phosphorylation as follows:

1. One possibility is that under cellular stress caused by p25 overexpression, autophagosomes containing various cytosolic proteins together with p25 may fuse with BACE1-containing endosomes, and p25/Cdk5-mediated BACE1 phosphorylation may then occur in the lumen of amphisomes. The autophagosomes can fuse with endocytic structures, such as multivesicular bodies (MVBs), generating the amphisome, which fuses with the lysosome to degrade the cytosolic materials [46]. In AD brains, autophagy abnormalities, including marked accumulation of autophagic vacuoles (AVs), recognized as Aβ-generating compartments [47, 48], and impaired maturation of AVs to lysosomes [49, 50] have been reported; these factors may contribute to the pathogenesis of AD.
2. The second possibility is that cytosolic p25 and Cdk5 pass through leaky endosomal membranes and phosphorylate BACE1 in the lumen of endosomes because increased Aβ_1–42_ in p25 TG mice may disrupt endosomal/lysosomal membrane impermeability and cause membrane leakage [51, 52].
3. Another possibility is that p25/Cdk5 may be localized inside small vesicles of multivesicular endosomes by some unknown mechanism, perhaps in a similar way as the sequestration of glycogen synthase kinase 3 (GSK3) from the cytosol into MVBs upon Wnt stimulation [53]. Elevated Aβ_1–42_ in p25 TG mice may cause leakage of p25/Cdk5-containing small vesicles, and p25/Cdk5 can then phosphorylate BACE1 in the lumen of multivesicular endosomes. Previous studies have shown that Aβ_1–42_ is localized predominantly to MVBs in the AD brain [54].
4. An alternative possibility is that p25/Cdk5-mediated BACE1 phosphorylation may occur in the cytosol during or after translation of the BACE1 protein on free ribosomes, as reported in a previous study in which Dyrk2 was shown to be phosphorylated before leaving the ribosome [55]. The N-terminal ER signal sequence may then target BACE1 to the ER, where BACE1 undergoes dimerization [31]. In fact, most of the phospho-BACE1 protein was found in the bottom fractions, where organelle markers of the ER, Golgi, endosomes, and lysosomes are localized.

From the results herein, we propose the following mechanism for the regulation of BACE1 activity in AD brains. Aberrant Cdk5 activation by p25 upregulates BACE activity through enhanced phosphorylation of BACE1, and this process contributes to the pathogenesis of sporadic AD. Furthermore, the elevated p25/Cdk5 levels could contribute to the phosphorylation of STAT3, which in turn transcriptionally upregulate both BACE1 [20] and γ-secretase component presenilin-1, and thereby increase the Aβ levels [56]. The role of p25/Cdk5 in the pathogenesis of AD, such as Aβ production, Tau pathology, neuronal loss, and cognitive and synaptic defects, has been established [20, 41, 57–60]. Our finding that p25/Cdk5 stimulates BACE1 activity supports that p25/Cdk5 may represent a promising target for the development of drugs to treat AD.

## References

[1] Vassar R, Bennett BD, Babu-Khan S, Kahn S, Mendiaz EA, Denis P, et al Beta-secretase cleavage of Alzheimer's amyloid precursor protein by the transmembrane aspartic protease BACE. Science 1999;286(5440):735–41. 10531052 10.1126/science.286.5440.735

[2] Fukumoto H, Rosene DL, Moss MB, Raju S, Hyman BT, Irizarry MC. Beta-secretase activity increases with aging in human, monkey, and mouse brain. Am J Pathol 2004;164(2):719–25. 14742275 10.1016/s0002-9440(10)63159-8 PMC1602259

[3] Apelt J, Bigl M, Wunderlich P, Schliebs R. Aging-related increase in oxidative stress correlates with developmental pattern of beta-secretase activity and beta-amyloid plaque formation in transgenic Tg2576 mice with Alzheimer-like pathology. Int J Dev Neurosci 2004;22(7):475–84. 15465277 10.1016/j.ijdevneu.2004.07.006

[4] Fukumoto H, Cheung BS, Hyman BT, Irizarry MC. Beta-secretase protein and activity are increased in the neocortex in Alzheimer disease. Arch Neurol 2002;59(9):1381–9. 12223024 10.1001/archneur.59.9.1381

[5] Holsinger RM, McLean CA, Beyreuther K, Masters CL, Evin G. Increased expression of the amyloid precursor beta-secretase in Alzheimer's disease. Ann Neurol 2002;51(6):783–6. 12112088 10.1002/ana.10208

[6] Sadleir KR, Vassar R. Cdk5 protein inhibition and Abeta42 increase BACE1 protein level in primary neurons by a post-transcriptional mechanism: implications of CDK5 as a therapeutic target for Alzheimer disease. J Biol Chem 2012;287(10):7224–35. 10.1074/jbc.M111.333914 22223639 PMC3293556

[7] Yang LB, Lindholm K, Yan R, Citron M, Xia W, Yang XL, et al Elevated beta-secretase expression and enzymatic activity detected in sporadic Alzheimer disease. Nat Med 2003;9(1):3–4. 12514700 10.1038/nm0103-3

[8] Harada H, Tamaoka A, Ishii K, Shoji S, Kametaka S, Kametani F, et al Beta-site APP cleaving enzyme 1 (BACE1) is increased in remaining neurons in Alzheimer's disease brains. Neurosci Res 2006;54(1):24–9. 16290302 10.1016/j.neures.2005.10.001

[9] Stockley JH, Ravid R, O'Neill C. Altered beta-secretase enzyme kinetics and levels of both BACE1 and BACE2 in the Alzheimer's disease brain. FEBS Lett 2006;580(28–29):6550–60. 17113083 10.1016/j.febslet.2006.10.076

[10] Leuba G, Wernli G, Vernay A, Kraftsik R, Mohajeri MH, Saini KD. Neuronal and nonneuronal quantitative BACE immunocytochemical expression in the entorhinohippocampal and frontal regions in Alzheimer's disease. Dement Geriatr Cogn Disord 2005;19(4):171–83. 15677864 10.1159/000083496

[11] Hebert SS, Horre K, Nicolai L, Papadopoulou AS, Mandemakers W, Silahtaroglu AN, et al Loss of microRNA cluster miR-29a/b-1 in sporadic Alzheimer's disease correlates with increased BACE1/beta-secretase expression. Proc Natl Acad Sci U S A 2008;105(17):6415–20. 10.1073/pnas.0710263105 18434550 PMC2359789

[12] Yasojima K, McGeer EG, McGeer PL. Relationship between beta amyloid peptide generating molecules and neprilysin in Alzheimer disease and normal brain. Brain Res 2001;919(1):115–21. 11689168 10.1016/s0006-8993(01)03008-6

[13] Preece P, Virley DJ, Costandi M, Coombes R, Moss SJ, Mudge AW, et al Beta-secretase (BACE) and GSK-3 mRNA levels in Alzheimer's disease. Brain Res Mol Brain Res 2003;116(1–2):155–8. 12941471 10.1016/s0169-328x(03)00233-x

[14] Stockley JH, O'Neill C. Understanding BACE1: essential protease for amyloid-beta production in Alzheimer's disease. Cell Mol Life Sci 2008;65(20):3265–89. 10.1007/s00018-008-8271-3 18695942 PMC11131673

[15] Dhavan R, Tsai LH. A decade of CDK5. Nat Rev Mol Cell Biol 2001;2(10):749–59. 11584302 10.1038/35096019

[16] Patrick GN, Zukerberg L, Nikolic M, de la Monte S, Dikkes P, Tsai LH. Conversion of p35 to p25 deregulates Cdk5 activity and promotes neurodegeneration. Nature 1999;402(6762):615–22. 10604467 10.1038/45159

[17] Tseng HC, Zhou Y, Shen Y, Tsai LH. A survey of Cdk5 activator p35 and p25 levels in Alzheimer's disease brains. FEBS Lett 2002;523(1–3):58–62. 12123804 10.1016/s0014-5793(02)02934-4

[18] Swatton JE, Sellers LA, Faull RL, Holland A, Iritani S, Bahn S. Increased MAP kinase activity in Alzheimer's and Down syndrome but not in schizophrenia human brain. Eur J Neurosci 2004;19(10):2711–9. 15147305 10.1111/j.0953-816X.2004.03365.x

[19] Tandon A, Yu H, Wang L, Rogaeva E, Sato C, Chishti MA, et al Brain levels of CDK5 activator p25 are not increased in Alzheimer's or other neurodegenerative diseases with neurofibrillary tangles. J Neurochem 2003;86(3):572–81. 12859671 10.1046/j.1471-4159.2003.01865.x

[20] Wen Y, Yu WH, Maloney B, Bailey J, Ma J, Marie I, et al Transcriptional regulation of beta-secretase by p25/cdk5 leads to enhanced amyloidogenic processing. Neuron 2008;57(5):680–90. 10.1016/j.neuron.2008.02.024 18341989 PMC2329816

[21] Kawarabayashi T, Younkin LH, Saido TC, Shoji M, Ashe KH, Younkin SG. Age-dependent changes in brain, CSF, and plasma amyloid (beta) protein in the Tg2576 transgenic mouse model of Alzheimer's disease. J Neurosci 2001;21(2):372–81. 11160418 10.1523/JNEUROSCI.21-02-00372.2001 PMC6763819

[22] Son MY, Chung SH. Expression of p25, an aberrant cyclin-dependent kinase 5 activator, stimulates basal secretion in PC12 cells. Mol Cells 2010;29(1):51–6. 10.1007/s10059-010-0016-0 20033852

[23] Chung SH, Takai Y, Holz RW. Evidence that the Rab3a-binding protein, rabphilin3a, enhances regulated secretion. Studies in adrenal chromaffin cells. J Biol Chem 1995;270(28):16714–8. 7622481 10.1074/jbc.270.28.16714

[24] Ahlijanian MK, Barrezueta NX, Williams RD, Jakowski A, Kowsz KP, McCarthy S, et al Hyperphosphorylated tau and neurofilament and cytoskeletal disruptions in mice overexpressing human p25, an activator of cdk5. Proc Natl Acad Sci U S A 2000;97(6):2910–5. 10706614 10.1073/pnas.040577797 PMC16029

[25] Seo H, Sonntag KC, Isacson O. Generalized brain and skin proteasome inhibition in Huntington's disease. Ann Neurol 2004;56(3):319–28. 15349858 10.1002/ana.20207

[26] Park JH, Jung MS, Kim YS, Song WJ, Chung SH. Phosphorylation of Munc18-1 by Dyrk1A regulates its interaction with Syntaxin 1 and X11alpha. J Neurochem 2012;122(5):1081–91. 10.1111/j.1471-4159.2012.07861.x 22765017

[27] Ryoo SR, Jeong HK, Radnaabazar C, Yoo JJ, Cho HJ, Lee HW, et al DYRK1A-mediated hyperphosphorylation of Tau. A functional link between Down syndrome and Alzheimer disease. J Biol Chem 2007;282(48):34850–7. 17906291 10.1074/jbc.M707358200

[28] Lee MS, Kao SC, Lemere CA, Xia W, Tseng HC, Zhou Y, et al APP processing is regulated by cytoplasmic phosphorylation. J Cell Biol 2003;163(1):83–95. 14557249 10.1083/jcb.200301115 PMC2173445

[29] Lin P, Le-Niculescu H, Hofmeister R, McCaffery JM, Jin M, Hennemann H, et al The mammalian calcium-binding protein, nucleobindin (CALNUC), is a Golgi resident protein. J Cell Biol 1998;141(7):1515–27. 9647645 10.1083/jcb.141.7.1515 PMC2132997

[30] Lee SY, Wenk MR, Kim Y, Nairn AC, De Camilli P. Regulation of synaptojanin 1 by cyclin-dependent kinase 5 at synapses. Proc Natl Acad Sci U S A 2004;101(2):546–51. 14704270 10.1073/pnas.0307813100 PMC327184

[31] Schmechel A, Strauss M, Schlicksupp A, Pipkorn R, Haass C, Bayer TA, et al Human BACE forms dimers and colocalizes with APP. J Biol Chem 2004;279(38):39710–7. 15247262 10.1074/jbc.M402785200

[32] Sidera C, Liu C, Austen B. Pro-domain removal in ASP-2 and the cleavage of the amyloid precursor are influenced by pH. BMC Biochem 2002;3:25 12204094 10.1186/1471-2091-3-25 PMC126479

[33] Marlow L, Cain M, Pappolla MA, Sambamurti K. Beta-secretase processing of the Alzheimer's amyloid protein precursor (APP). J Mol Neurosci 2003;20(3):233–9. 14501002 10.1385/JMN:20:3:233

[34] Iijima K, Ando K, Takeda S, Satoh Y, Seki T, Itohara S, et al Neuron-specific phosphorylation of Alzheimer's beta-amyloid precursor protein by cyclin-dependent kinase 5. J Neurochem 2000;75(3):1085–91. 10936190 10.1046/j.1471-4159.2000.0751085.x

[35] Westmeyer GG, Willem M, Lichtenthaler SF, Lurman G, Assfalg-Machleidt I, Reiss K, et al Dimerization of BACE. J Biol Chem 2004;279:53205–12. 15485862 10.1074/jbc.M410378200

[36] Hong L, Koelsch G, Lin X, Wu S, Terzyan S, Ghosh AK, et al Structure of the protease domain of memapsin 2 (beta-secretase) complexed with inhibitor. Science 2000;290(5489):150–3. 11021803 10.1126/science.290.5489.150

[37] Lee HW, Seo HS, Ha I, Chung SH. Overexpression of BACE1 stimulates spontaneous basal secretion in PC12 cells. Neurosci Lett 2007;421(2):178–83. 17566645 10.1016/j.neulet.2007.01.082

[38] Huse JT, Pijak DS, Leslie GJ, Lee VM, Doms RW. Maturation and endosomal targeting of beta-site amyloid precursor protein-cleaving enzyme. The Alzheimer's disease beta-secretase. J Biol Chem 2000;275(43):33729–37. 10924510 10.1074/jbc.M004175200

[39] Sun X, Bromley-Brits K, Song W. Regulation of beta-site APP-cleaving enzyme 1 gene expression and its role in Alzheimer's disease. J Neurochem 2012;120 Suppl 1:62–70. 10.1111/j.1471-4159.2011.07515.x 22122349

[40] Sultana R, Butterfield DA. Regional expression of key cell cycle proteins in brain from subjects with amnestic mild cognitive impairment. Neurochem Res 2007;32(4–5):655–62. 17006763 10.1007/s11064-006-9123-x

[41] Giusti-Rodriguez P, Gao J, Graff J, Rei D, Soda T, Tsai LH. Synaptic deficits are rescued in the p25/Cdk5 model of neurodegeneration by the reduction of beta-secretase (BACE1). J Neurosci 2011;31(44):15751–6. 10.1523/JNEUROSCI.3588-11.2011 22049418 PMC3248793

[42] Snow AD, Mar H, Nochlin D, Sekiguchi RT, Kimata K, Koike Y, et al Early accumulation of heparan sulfate in neurons and in the beta-amyloid protein-containing lesions of Alzheimer's disease and Down's syndrome. Am J Pathol 1990;137(5):1253–70. 2146882 PMC1877656

[43] Perry G, Siedlak SL, Richey P, Kawai M, Cras P, Kalaria RN, et al Association of heparan sulfate proteoglycan with the neurofibrillary tangles of Alzheimer's disease. J Neurosci 1991;11(11):3679–83. 1941102 10.1523/JNEUROSCI.11-11-03679.1991 PMC6575552

[44] Cataldo AM, Peterhoff CM, Troncoso JC, Gomez-Isla T, Hyman BT, Nixon RA. Endocytic pathway abnormalities precede amyloid beta deposition in sporadic Alzheimer's disease and Down syndrome: differential effects of APOE genotype and presenilin mutations. Am J Pathol 2000;157(1):277–86. 10880397 10.1016/s0002-9440(10)64538-5 PMC1850219

[45] Cataldo AM, Petanceska S, Terio NB, Peterhoff CM, Durham R, Mercken M, et al Abeta localization in abnormal endosomes: association with earliest Abeta elevations in AD and Down syndrome. Neurobiol Aging 2004;25(10):1263–72. 15465622 10.1016/j.neurobiolaging.2004.02.027

[46] Fader CM, Colombo MI. Autophagy and multivesicular bodies: two closely related partners. Cell Death Differ 2009;16(1):70–8. 10.1038/cdd.2008.168 19008921

[47] Yu WH, Cuervo AM, Kumar A, Peterhoff CM, Schmidt SD, Lee JH, et al Macroautophagy—a novel Beta-amyloid peptide-generating pathway activated in Alzheimer's disease. J Cell Biol 2005;171(1):87–98. 16203860 10.1083/jcb.200505082 PMC2171227

[48] Yu WH, Kumar A, Peterhoff C, Shapiro Kulnane L, Uchiyama Y, Lamb BT, et al Autophagic vacuoles are enriched in amyloid precursor protein-secretase activities: implications for beta-amyloid peptide over-production and localization in Alzheimer's disease. Int J Biochem Cell Biol 2004;36(12):2531–40. 15325590 10.1016/j.biocel.2004.05.010

[49] Nixon RA, Wegiel J, Kumar A, Yu WH, Peterhoff C, Cataldo A, et al Extensive involvement of autophagy in Alzheimer disease: an immuno-electron microscopy study. J Neuropathol Exp Neurol 2005;64(2):113–22. 15751225 10.1093/jnen/64.2.113

[50] Nixon RA, Yang DS. Autophagy failure in Alzheimer's disease—locating the primary defect. Neurobiol Dis 2011;43(1):38–45. 10.1016/j.nbd.2011.01.021 21296668 PMC3096679

[51] Ditaranto K, Tekirian TL, Yang AJ. Lysosomal membrane damage in soluble Abeta-mediated cell death in Alzheimer's disease. Neurobiol Dis 2001;8(1):19–31. 11162237 10.1006/nbdi.2000.0364

[52] Yang AJ, Chandswangbhuvana D, Margol L, Glabe CG. Loss of endosomal/lysosomal membrane impermeability is an early event in amyloid Abeta1-42 pathogenesis. J Neurosci Res 1998;52(6):691–8. 9669318 10.1002/(SICI)1097-4547(19980615)52:6<691::AID-JNR8>3.0.CO;2-3

[53] Taelman VF, Dobrowolski R, Plouhinec JL, Fuentealba LC, Vorwald PP, Gumper I, et al Wnt signaling requires sequestration of glycogen synthase kinase 3 inside multivesicular endosomes. Cell 2010;143(7):1136–48. 10.1016/j.cell.2010.11.034 21183076 PMC3022472

[54] Takahashi RH, Milner TA, Li F, Nam EE, Edgar MA, Yamaguchi H, et al Intraneuronal Alzheimer abeta42 accumulates in multivesicular bodies and is associated with synaptic pathology. Am J Pathol 2002;161(5):1869–79. 12414533 10.1016/s0002-9440(10)64463-x PMC1850783

[55] Lochhead PA, Sibbet G, Morrice N, Cleghon V. Activation-loop autophosphorylation is mediated by a novel transitional intermediate form of DYRKs. Cell 2005;121(6):925–36. 15960979 10.1016/j.cell.2005.03.034

[56] Liu L, Martin R, Chan C. Palmitate-activated astrocytes via serine palmitoyltransferase increase BACE1 in primary neurons by sphingomyelinases. Neurobiol Aging 2013;34(2):540–50. 10.1016/j.neurobiolaging.2012.05.017 22727944 PMC3459302

[57] Noble W, Olm V, Takata K, Casey E, Mary O, Meyerson J, et al Cdk5 is a key factor in tau aggregation and tangle formation in vivo. Neuron 2003;38(4):555–65. 12765608 10.1016/s0896-6273(03)00259-9

[58] Cruz JC, Tseng HC, Goldman JA, Shih H, Tsai LH. Aberrant Cdk5 activation by p25 triggers pathological events leading to neurodegeneration and neurofibrillary tangles. Neuron 2003;40(3):471–83. 14642273 10.1016/s0896-6273(03)00627-5

[59] Cruz JC, Kim D, Moy LY, Dobbin MM, Sun X, Bronson RT, et al p25/cyclin-dependent kinase 5 induces production and intraneuronal accumulation of amyloid beta in vivo. J Neurosci 2006;26(41):10536–41. 17035538 10.1523/JNEUROSCI.3133-06.2006 PMC6674706

[60] Fischer A, Sananbenesi F, Pang PT, Lu B, Tsai LH. Opposing roles of transient and prolonged expression of p25 in synaptic plasticity and hippocampus-dependent memory. Neuron 2005;48(5):825–38. 16337919 10.1016/j.neuron.2005.10.033

